# A Comparison of Constitutive and Inducible Non-Endogenous Keto-Carotenoids Biosynthesis in *Synechocystis* sp. PCC 6803

**DOI:** 10.3390/microorganisms7110501

**Published:** 2019-10-28

**Authors:** Barbara Menin, Andrea Lami, Simona Musazzi, Anastasia A. Petrova, Stefano Santabarbara, Anna Paola Casazza

**Affiliations:** 1 Istituto di Biologia e Biotecnologia Agraria, Consiglio Nazionale delle Ricerche, Via Bassini 15a, 20133 Milano, Italy; 2 Photosynthesis Research Unit, Consiglio Nazionale delle Ricerche, Via Celoria 26, 20133 Milano, Italy; 3 Istituto di Ricerca sulle Acque-Verbania, Consiglio Nazionale delle Ricerche, Largo Tonolli 50, 28933 Verbania, Italy

**Keywords:** astaxanthin, canthaxanthin, keto-carotenoids, metabolic engineering, *Synechocystis* sp. PCC 6803

## Abstract

The model cyanobacterium *Synechocystis* sp. PCC 6803 has gained significant attention as an alternative and sustainable source for biomass, biofuels and added-value compounds. The latter category includes keto-carotenoids, which are molecules largely employed in a wide spectrum of industrial applications in the food, feed, nutraceutical, cosmetic and pharmaceutical sectors. Keto-carotenoids are not naturally synthesized by *Synechocystis*, at least in any significant amounts, but their accumulation can be induced by metabolic engineering of the endogenous carotenoid biosynthetic pathway. In this study, the accumulation of the keto-carotenoids astaxanthin and canthaxanthin, resulting from the constitutive or temperature-inducible expression of the *CrtW* and *CrtZ* genes from *Brevundimonas*, is compared. The benefits and drawbacks of the two engineering approaches are discussed.

## 1. Introduction

The global carotenoids market size, mainly covered by β-carotene (β-car), lutein, lycopene, astaxanthin (Asx), zeaxanthin (Zea) and canthaxanthin (Can), is estimated to nearly double over the next six years [1], with Asx, and to a lesser extent Can, showing the greatest exponential, growth [2]. Asx has a wide application as a pigmentation source in aquaculture [3] and poultry farming [4] and its usage for food and beverage fortification, nutraceutical and cosmetic formulations is steadily increasing [5,6]. At the moment, synthetic sources are dominating the overall industry, but natural ones are expected to witness significant gains due to growing consumer preference and demand for more eco-friendly products and processes. Moreover, the rising consumer awareness regarding health benefits and advantages associated with natural antioxidants consumption [7] will further boost this market segment. The principal natural sources for the industrial production of Asx are the green alga *Haematococcus pluvialis* [8] and the heterobasidiomycetous yeast *Xanthophyllomyces dendrorhous* [9]. *H. pluvialis* produces by far the highest amount of Asx, around 40 mg/g dry cell weight (DCW), but mainly in its esterified form, thus reducing its application potential in drug development. Moreover, long two-stage culture periods are required and the extraction procedure is complicated by the presence of chlorophyll and fatty acids. Conversely, *X. dendrorhous* synthesises essentially non-esterified Asx, but the expensive fermentation procedure hinders large-scale production from this organism. These drawbacks, together with biosafety issues related to chemical synthesis, make the microbial production of Asx an attractive alternative. Since the pioneering work of Misawa and colleagues using *Escherichia coli* as recombinant host [10], the number of reports on the production of Asx in non-carotenogenic microorganisms, like *Saccharomyces cerevisiae* and *E. coli*, is constantly increasing. Common to these studies is the introduction of key enzymes of the Asx biosynthetic pathway from different species. The level of Asx accumulation in these organisms is, however, generally lower than in *H. pluvialis*, ranging from 4.7 to 8.1 mg/g DCW in *S. cerevisiae* [11,12] and up to 8.3 mg/g DCW in *E. coli* [13,14,15], but their high growth rate may, in principle, compensate for lower production. Cyanobacteria, in particular the model species *Synechocystis* sp. PCC 6803 and *Synechococcus* sp. PCC 7942 and PCC 7002, are good candidates for becoming the “green *E. coli*” [16], due to their relatively fast growth rate, their genetic malleability and also thanks to the availability of several genetic and synthetic biological tools for their manipulation [17,18]. Cyanobacteria are thus receiving considerable attention as promising alternative platforms for the production of biofuels and various industrially relevant chemicals, as well as molecules of higher value [19,20]. 

The first and, to our knowledge, only report on transgenic model cyanobacteria producing Asx is dated back to 1997. By inserting the *CrtO* gene from *H. pluvialis*, encoding a 4,4′ β-car oxygenase which converts β-car to Can, a small amount of Asx and Can (~3% and ~1% of total carotenoids, respectively), as well as traces of other intermediates of the Asx biosynthetic pathway, could be detected in the engineered *Synechococcus* sp. PCC 7942 strain [21]. We recently showed that in *Synechocystis* sp. PCC 6803, the introduction of an expression plasmid carrying the 4,4′ β-car oxygenase (*CrtW*) and the 3,3′ β-car hydroxylase (*CrtZ*) genes from the proteobacterium *Brevundimonas* sp. SD-212 under the control of a temperature-inducible promoter, allowed the accumulation of consistent amounts of Asx, which could represent up to 50% of the total carotenoids [22]. Here we present an alternative metabolic engineering approach, in which the same carotenogenic enzymes CrtW and CrtZ from *Brevundimonas* sp. SD-212 are integrated into the genome of *Synechocystis* sp. PCC 6803 and constitutively expressed under the control of a strong endogenous promoter. The productivity of the constitutively expressing strains is compared with the one attained by the temperature-inducible ones, where the expression of CrtW and CrtZ has been appropriately tuned by shifting the cultures to increasing temperatures up to 39 °C. Both engineering approaches were successful, but significant differences in terms of end-product accumulation levels and relative abundance of endogenous and non-endogenous ketolated and hydroxylated carotenoids were observed. Advantages and disadvantages of the two strategies are discussed also concerning the potential exploitation of these strains for the production of added-value compounds.

## 2. Materials and Methods

### 2.1. Bacterial Strains and Growth Conditions

The glucose-tolerant wild-type *Synechocystis* sp. PCC 6803 strain (hereafter *Synechocystis*) was obtained from Peter J. Nixon, Imperial College, London, UK. *Synechocystis* temperature-inducible (TI) strains (TI-W, TI-Z, TI-WZ and TI-ZW) were already available ([22], therein referred to as S6803W, S6803Z, S6803WZ and S6803ZW, respectively). These strains carry the exogenous *CrtW* and *CrtZ* genes cloned in the conditional, temperature-controlled, expression vector pFC1 [23]. The generation of *Synechocystis* strains constitutively (C) expressing the exogenous *CrtW* and *CrtZ* genes (C-W, C-Z, C-WZ and C-ZW) is described below. Wild-type and engineered *Synechocystis* strains were routinely grown at 28 °C in liquid BG-11 medium under continuous white light (20 μmol of photons m^−2^ s^−1^ provided by L36W/840 fluorescent lamps, OSRAM GmbH, Munich, Germany). Strains were also maintained on solid BG-11 medium, supplemented with 10 mM TES and long-term cryopreserved in 8% DMSO at −80 °C. Heat induction experiments were performed by shifting exponentially growing cultures from 28 °C to 33 °C and 39 °C for 2 days. Experiments were repeated three times with all strains. Samples were collected by centrifugation (12,000× *g* for 10 min at room temperature) or by vacuum filtration (depending on culture volumes) and immediately frozen in liquid nitrogen. Samples were stored at −80 °C until pigments analysis was performed. For gene manipulation, the *Escherichia coli* strain DH5α was used. All media were supplemented with appropriate antibiotics as required: for *E. coli*, ampicillin (Ap) at 100 μg mL^−1^; and for *Synechocystis*, kanamycin (Km) at 40 μg mL^−1^ for constitutively expressing strains and streptomycin (Sm) at 5 μg mL^−1^ in combination with spectinomycin (Sp) at 5 μg mL^−1^ for temperature-inducible strains.

### 2.2. Plasmid Construction for Chromosomal Integration

Synthetic sequences, delivered in pUC57 (pUC57-Syn_CrtW and pUC57-Syn_CrtZ), encoding the β-carotene ketolase CrtW and the β-carotene hydroxylase CrtZ from *Brevundimonas* sp. SD212, optimised accordingly to the codon usage of *Synechocystis* (GenBank accession numbers MK214312 and MK214313, respectively) and flanked by three restriction sites (NdeI at the 5′ end and XhoI/NdeI at the 3′ end) and a 3′-ribosome binding site, were the same as in [22]. The coding sequences of *CrtW* and *CrtZ* were amplified from pUC57-Syn_CrtW and pUC57-Syn_CrtZ by PCR using primers (Table S1) carrying the restriction sites for NotI at the 5′ end and for BglII at the 3′ end (New England Biolabs, Ipswich, MA, USA). After NotI/BglII cleavage, PCR products were cloned into the pPD-FLAG-Km plasmid (kindly provided by Roman Sobotka), yielding pPD-FCrtW and pPD-FCrtZ (Figure S1). pPD-FLAG-Km contains the *Synechocystis psbAII* promoter, a sequence encoding the 3 × FLAG tag and flanking sequences for homologous recombination that allows the insertion of tagged constructs into the *Synechocystis* genome in place of the *psbAII* gene [24]. For tandem gene cloning, the sequences comprising the *CrtW* and *CrtZ* coding region and the 3′-ribosome binding site were excised from pUC57-Syn_CrtW and pUC57-CrtZ with NdeI and cloned into pPD-FCrtZ and pPD-FCrtW, respectively, both opened with the same enzyme, yielding pPD-CrtW-FCrtZ and pPD-CrtZ-FCrtW (Figure S1). All constructs were verified by nucleotide sequencing (BigDye Sequencing Kit, Applied Biosystems Inc., CA, USA). 

### 2.3. Synechocystis Transformation

A small amount of *Synechocystis* cells was collected with a loop from two-week-old BG-11 agar plates and resuspended in 100 μL of BG-11. After the addition of 750 ng of circular plasmid DNA, cells were incubated at 28 °C under moderately high light (100 μmol of photons m^−2^ s^−1^) for 1–3 h and then gently spread on BG-11 agar plates lacking antibiotics. Once dried, plates were incubated overnight in continuous light (20 μmol of photons m^−2^ s^−1^) at 28 °C. Cells were then transferred to BG-11 agar plates supplemented with 10 μg mL^−1^ Km and evenly spread on the entire surface. Plates were incubated under continuous normal light (20 μmol of photons m^−2^ s^−1^) at 28 °C until the culture died and transformed colonies appeared. Single colonies were picked, streaked on BG-11 plates containing a higher amount of Km (20 μg mL^−1^) and analysed by PCR (primers used are listed in Table S1) to verify the successful integration of the exogenous *CrtW* and *CrtZ* genes at the *psbAII* locus. Positive colonies were repeatedly sub-cultured (for at least four rounds) onto BG-11 plates containing 40 μg mL^−1^ Km to promote segregation.

### 2.4. Absorption Spectroscopy

Whole cells absorption spectra were recorded in the 400–760 nm interval using a V630 spectrophotometer (Jasco Europe, Cremella LC, Italy) at a scan speed of 100 nm min^−1^ on freshly collected samples diluted to an optical density at 680 nm of ~0.4 with BG-11. In order to compensate for light scattering of the cell suspension, opal glasses were placed between the sample and the light detector of the spectrophotometer. Measurements were performed twice on three independent biological replicates.

### 2.5. Pigments Extraction and Analysis

Pigments were extracted overnight in the dark, with 90% acetone, under nitrogen. The acetone extract was cleared by centrifugation (3400× *g* for 10 min) and the supernatant was filtered through 0.45 µm syringe filter. Pigment determination was performed by HPLC following [25] and using an HPLC system (Thermo Scientific UltiMate 3000, Thermo Fisher Scientific, Waltham, MA, USA) consisting of an autosampler, a quaternary pump (P680), a thermostatted column oven (TCC100) and a DAD detector (DAD 3000RS). The column was an ODS (C18) column (Omnispher, length 250 mm, 4.6 mm ID, 5µm particle size). Chromatograms were acquired at 460 nm and 656 nm for carotenoids and chlorophylls, respectively. For peak identification, the 380–800 nm spectra were also recorded. After sample injection (100 µL of acetone extract), a gradient program that ramped from 85% mobile-phase A (80:20, *vol*/*vol*, methanol: aqueous solution of 0.001 M PIC A ion-pairing and 0.001 M propionic acid) to 100% mobile-phase B (60:40, acetone: methanol) in 30 min with a hold for 20 min provided sufficient resolution of all pigments of interest. Flow rates were from 1 mL min^−1^ to 2 mL min^−1^. Between samples, the column was re-equilibrated by linear ramping to 85% mobile-phase A for 5 min and maintenance for 10 min. The quantification was based on calibration with standards form DHI (Hørsholm, Denmark). Pigment concentrations were determined on the basis of molar extinction coefficients at the detection wavelengths. The specific extinction coefficients E^1%^_(460 nm)_ and E^1%^_(656 nm)_ were derived from the E^1%^ max reported in [26,27]. Measurements were performed twice on three independent biological replicates.

## 3. Results

### 3.1. Generation of Synechocystis Engineered Strains Constitutively Expressing Exogenous CrtW and CrtZ

In order to obtain an “always on” expression of the carotenogenic enzymes CrtW and CrtZ from *Brevundimonas* sp. SD-212 in *Synechocystis*, the strong native promoter *psbAII* was chosen [28]. Thus, the synthetic genes *CrtW* and *CrtZ*, codon optimised for *Synechocystis* [22], were cloned in the transformation vector pPD-FLAG-Km [24], which targets the *psbAII* gene site in the *Synechocystis* genome. Genes were cloned singularly or in tandem with a ribosome binding site in between. The resulting plasmids were used for *Synechocystis* transformation, yielding the constitutively expressing strains (C-strains) C-W, C-Z, C-WZ and C-ZW. The plasmid for the expression of CrtZ, alone, generated significantly more transformants than the others (at least ten-fold more), suggesting that the observed low efficiency could be due to the expression of CrtW. Moreover, despite repeated sub-cloning of the engineered strains on selective media, full segregation could not be attained.

### 3.2. Constitutive Expression of Exogenous CrtW and CrtZ

**Figure 1:**
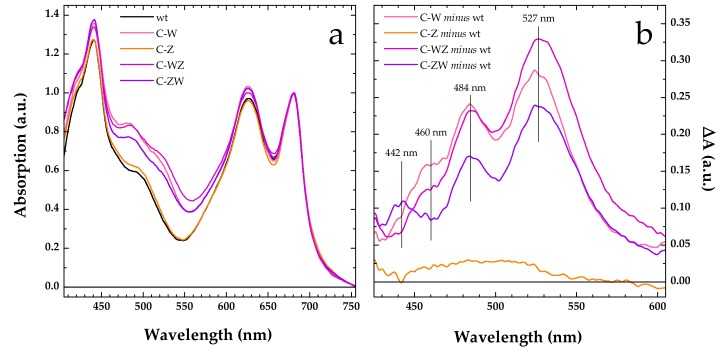
Whole cells absorption spectra. (**a**) Comparison of absorption spectra of wild-type (wt, black line) and engineered *Synechocystis* strains constitutively expressing *Brevundimonas* CrtW (C-W, pink line), CrtZ (C-Z, orange line), CrtW and CrtZ in tandem (C-WZ, purple line), CrtZ and CrtW in tandem (C-ZW, violet line). All spectra are normalised at their maximal Qy absorbance (680 nm). (**b**) Comparison of the difference spectra “engineered strain *minus* wild-type” in the 425–605 nm window, colour coding as for the engineered strains in panel (**a**). The main spectral features of the difference spectra are marked by vertical solid lines and the wavelength indicated.

Already during transformants selection, the engineered *Synechocystis* strains C-W, C-Z, C-WZ and C-ZW, which were expected to constitutively produce the exogenous CrtW and CrtZ enzymes, could be clearly distinguished from the wild-type by simply observing the colour of the cells. When compared with the wild-type, the C-Z strain showed a slightly more brilliant shade of green, while the colour of all the other strains markedly leaned to moss-green (Figure S2). Moreover, the engineered strains expressing CrtW, alone or in combination with CrtZ, displayed a longer doubling time (~24 h) with respect to the wild-type and the C-Z strain, both doubling in ~16 h. The altered colour observed in the engineered strains was further confirmed by measuring whole cells absorption spectra (Figure 1a). All spectra were nearly identical above 660 nm, where the absorption is dominated by Chlorophyll *a*, and only small variations in intensity could be found in the 600–650 nm interval, linked to slight differences in the phycobilisomes content. Much more pronounced was instead the spectral variation associated to a broadening in the carotenoid absorption region (450–570 nm). This was particularly evident in the strains expressing CrtW (C-W, C-WZ and C-ZW). As highlighted by the difference (engineered strain *minus* wild-type) spectra shown in Figure 1b, the main spectral changes occurred at 527 and 484 nm, with a shoulder centred at around 460 nm in both C-W and C-WZ, whereas in C-ZW three distinct features were detected at 527, 484 and 442 nm. On the contrary, the absorption spectrum of the C-Z strain, expressing only CrtZ, overlapped to a large extent with the one of the wild-types (Figure 1a), showing only a small, spectrally unstructured, difference covering the 450–550 nm window (Figure 1b). These results were fully consistent with what was previously observed in the temperature-inducible (TI) strains [22], confirming that the constitutive expression of the same exogenous *CrtW* and *CrtZ* genes also led to significant changes in the carotenoid composition of the engineered strains.

**Figure 2:**
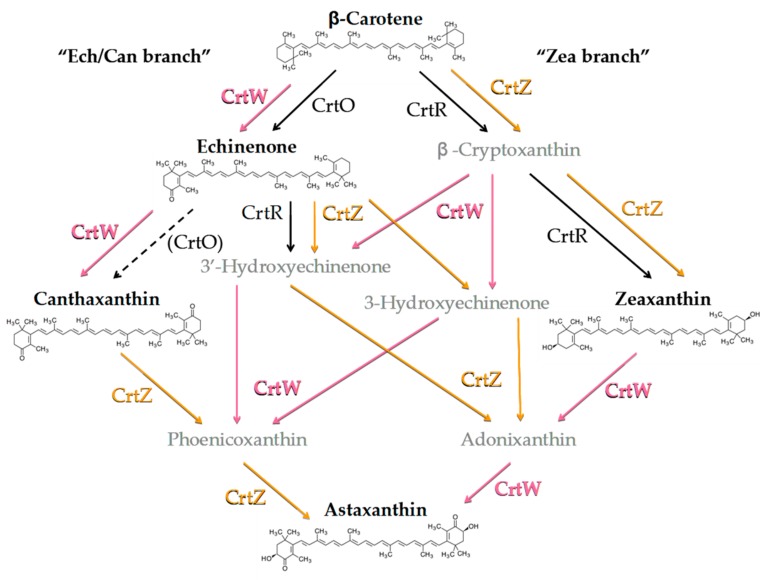
Simplified scheme of the astaxanthin biosynthetic pathway from endogenous β-carotene (β-car) in engineered *Synechocystis* strains, resulting from the heterologous expression of *Brevundimonas* CrtW and CrtZ. The endogenous enzymes CrtO (β-car ketolase) and CrtR (β-car hydroxylase) are shown in black. Parentheses and the dashed arrow indicate weak or possible catalytic function. *Brevundimonas* β-car ketolase (CrtW) and β-car hydroxylase (CrtZ) are indicated in pink and orange letters, respectively. Ech, echinenone; Can, canthaxanthin; Zea, zeaxanthin.

In order to investigate the carotenoids profile of the engineered strains in greater detail, total pigments were extracted and analysed by HPLC. The analysis was focussed on Asx, its precursor β-car and the main intermediates of the biosynthetic pathway (Figure 2), namely echinenone (Ech), Can and Zea. 

**Figure 3:**
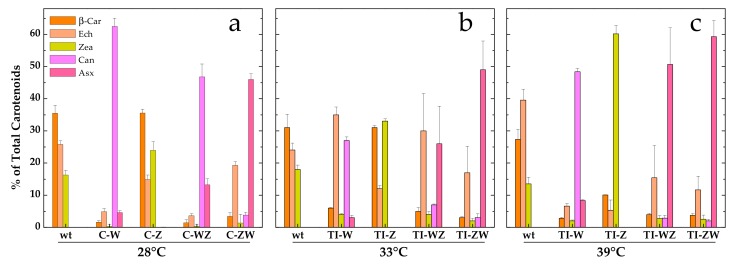
Relative distribution of the main carotenoids in the different engineered *Synechocystis* strains with respect to the wild-type. (**a**) Engineered strains constitutively expressing *Brevundimonas* CrtW and CrtZ (C-W, C-Z, C-WZ and C-ZW) grown at 28 °C. (**b**) and (**c**) Engineered strains expressing *Brevundimonas* CrtW and CrtZ in a temperature-controlled manner (TI-W, TI-Z, TI-WZ and TI-ZW) after two days induction at (**b**) 33 °C or (**c**) 39 °C. β-car, β-carotene (orange); Ech, echinenone (peach); Zea, zeaxanthin (yellow); Can, canthaxanthin (light magenta); Asx, astaxanthin (pink). wt, wild-type. Error bars indicate SE.

As shown in Figure 3a, the C-Z strain, constitutively expressing only CrtZ, synthesised the same carotenoids as the wild-type but in different proportions with Zea being more abundant than Ech. In line with what was observed in the whole cell absorption spectra (Figure 1), more evident changes in the carotenoid composition were obtained for all other strains. The C-W strain, constitutively expressing only CrtW, accumulated almost exclusively Can (~60%, corresponding to 1.3 ± 0.1 mg/g DCW) as well as some Asx (~5%, 0.09 ± 0.02 mg/g DCW), with β-car and Ech accounting together for much less than 10% of the total carotenoids. Zea was nearly undetectable in this strain. C-WZ, which expresses both CrtW and CrtZ, also produced high amounts of Can (~45%, 0.9 ± 0.1 mg/g DCW), again at the expense of β-car, Ech and Zea, but synthesised higher amounts of Asx, being greater than 10% (0.26 ± 0.05 mg/g DCW) of the carotenoid pool. C-ZW, which also expresses both carotenogenic enzymes, was the most efficient strain in terms of Asx accumulation, which reached ~45% (0.92 ± 0.05 mg/g DCW) of the total carotenoids. Can, Zea and β-car were present in low amounts, while Ech was the second most abundant carotenoid in C-ZW (~20%). With the exception of C-Z, in all other strains the synthesis of the two keto-carotenoids Asx and Can was always linked to a significant reduction of the β-car and the Zea levels, while Ech was somewhat less affected, at least in the C-ZW strain (Figure 3a).

### 3.3. Temperature Induction of Exogenous CrtW and CrtZ

**Figure 4:**
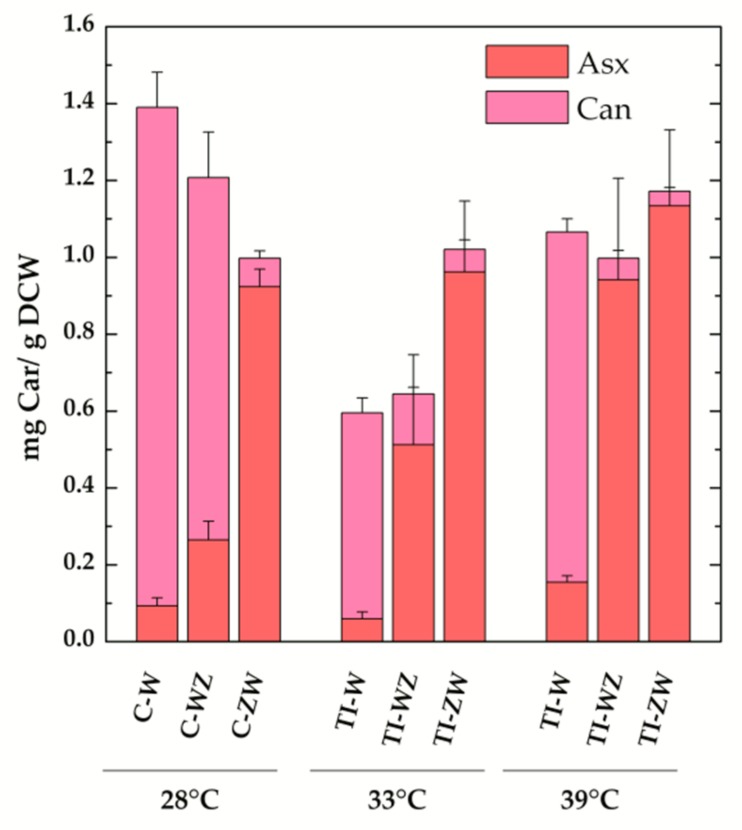
Absolute amounts of total exogenous keto-carotenoids in the engineered constitutive (C-) and temperature-inducible (TI-) *Synechocystis* strains. Also shown is the individual contribution of the carotenoids (Car) canthaxanthin (Can, light magenta) and astaxanthin (Asx, pink) to the total. Values are expressed as mg Car/g dry cell weight (DCW). Error bars indicate SE.

The TI-strains generated in [22] allow to investigate the temperature-controlled expression of the carotenogenic CrtW and CrtZ from *Brevundimonas* in *Synechocystis*. As reported in [22], by shifting the TI-strains to 33 °C, a value slightly above the optimal growth temperature for this organism, the synthesis of exogenous CrtW and CrtZ was induced, leading to a significant production of the non-endogenous keto-carotenoids Asx and Can (data from [22] are replotted in Figure 3b for ease of comparison). With the attempt to further increase the accumulation levels of Asx and Can, the TI-strains (TI-W, TI-Z, TI-WZ and TI-ZW) were initially grown at 28 °C, i.e., the standard growth condition under which CrtW and CrtZ are not produced, and then transferred to 39 °C for two days. At this temperature, which approaches the upper heat tolerance limit for *Synechocystis*, the expression level of CrtW and CrtZ was expected to be close to the maximal attainable [23,29,30]. Total pigments were extracted from 39 °C-induced cultures and analysed by HPLC, again with a particular focus on Asx and its biosynthetic precursors (Figure 3c). In the wild-type strain at 39 °C, the dominant carotenoid was Ech, followed by β-car and Zea. In the TI-Z strain, where only the expression of CrtZ was induced, a very considerable accumulation of Zea was obtained, which reached ~60% of the total carotenoid content, while β-car and Ech dropped to about 10% and 5%, respectively. When only the expression of the *CrtW* gene was induced (TI-W strain), the main carotenoid was Can (~50%, 0.91 ± 0.03 mg/g DCW), followed by Asx, Ech, β-car and Zea, all present in low amounts (<10%). After induction at 39 °C, the two strains expressing both CrtW and CrtZ, showed a very similar carotenoid profile, resembling the one of the TI-ZW strain upon induction at 33 °C (Figure 3b). Both strains accumulated significant levels of Asx (>50%, 0.9–1.1 mg/g DCW), with TI-ZW being slightly more efficient in producing Asx with a concomitant reduction of Ech. A summary of the absolute quantification of the exogenous keto-carotenoids accumulated in both the C- and the TI-strains is shown in Figure 4.

## 4. Discussion

Several factors have to be taken into account when performing metabolic engineering in *Synechocystis,* as well as in any other host organism, in order to reach the maximal accumulation of the molecule of interest and, at the same time, ensuring that none of the heterologous biosynthetic pathway products are potentially toxic for the organism, thereby maintaining cell viability.

A key step in optimising biosynthetic production is the identification of the most efficient enzymes to be introduced and, if necessary, the optimisation of their coding sequences to maximise gene expression. For the generation of the TI-strains and the C-strains here described, the carotenogenic enzymes CrtW and CrtZ from *Brevundimonas* sp. SD212 were chosen as these were reported to enable high production of Asx in plant as well as microbial hosts [31], with their sequences adapted to *Synechocystis* codon bias. 

Potential detrimental effects caused by exogenous biosynthetic products or redistribution/alteration of the endogenous metabolites can be tested and eventually attenuated by choosing the appropriate genetic modification approach. By using a shuttle vector carrying a temperature-inducible promoter for generating the TI-strains and an integrative vector harbouring a strong constitutive promoter for the C-strains, two fundamentally different scenarios were obtained. The first can be considered as the “safe one”, since the adopted system [23] allows for the switching on and fine tuning of the expression of the exogenous genes by simply changing the growth temperature once an adequate biomass production is attained. This is a non-negligible point if one considers that the eventual toxicity of the exogenous genes and/or the synthesised non-endogenous keto-carotenoids to the host cell is not predictable a priori. The second scenario constitutes the “risky one” since, in these strains, *CrtW* and *CrtZ* are expected to be highly transcribed in an ongoing manner, allowing, in principle, the continual production of substantial amounts of Asx and Can. Thus, the behaviour of the two sets of engineered strains will help in identifying weaknesses and strengths of the two strategies, defining the most promising one for successful Asx production in *Synechocystis*. 

Although all engineered strains showed significant alterations in the carotenoids profile (Figure 3), pronounced differences were detected depending on both the biosynthetic enzymes and their expression strategy. For instance, the CrtZ-expressing strains appeared to have, in general, the less affected carotenoid pattern, resulting only in a redistribution of the main endogenous carotenoids. The constitutive expression of CrtZ, despite a high level of transcript accumulation (Figure S3), led only to a small increase in the Zea content (a ~30% increment with respect to the wild-type), which almost perfectly matched the reduction in the Ech level, while the β-car pool remained unchanged (Figure 3a). A more significant enhancement in the Zea content was instead observed in the temperature-inducible strain (TI-Z) after incubation at 33 °C (Figure 3b), which represents an intermediate level of CrtZ expression. A further increase in Zea accumulation was obtained after shifting the culture to 39 °C (Figure 3c), i.e., when the expression of CrtZ is close to its maximum according to the robustly characterised promoter system response [23,29,30]. Under these conditions Zea became the dominant carotenoid, representing about 60% of the pool. Under constitutive expression and for intermediate expression at 33 °C, the introduction of the exogenous CrtZ appeared to re-distribute the proportions of the carotenoids derived from the β-car precursor in favour of Zea at a net loss of Ech. On the other hand, for the induction at 39 °C, both β-car and Ech were severely reduced with respect to the wild-type, for which Ech was the main carotenoid under these growth conditions. Thus the “Zea branch” of the pathway (Figure 2) became largely dominant in TI-Z at 39 °C (Figure 3c). The lower level of Zea accumulation in C-Z with respect to TI-Z (39°C) might then indicate a possible detrimental effect of long-term β-car depletion, whose biological function cannot be compensated by Zea replacement during growth but might be tolerated for the short-term induction period after the cultures reach the stationary phase.

In the strain stably expressing CrtW (C-W), Can became the principle carotenoid (Figure 3a), exceeding 60% of the total pool, indicating that most of the β-car precursor was diverted towards the “Ech/Can branch” of the pathway (Figure 2). Small amounts of Asx were observed as well (~5%) concomitantly with the almost complete suppression of Zea accumulation. Surprisingly, the RT-PCR analysis indicated a rather low level of *CrtW* transcript (Figure S3) despite the gene being under the control of the same strong promoter as *CrtZ* in C-Z. Hence in the single-gene-expressing C-strains, the accumulation of exogenous keto-carotenoids appears to be largely uncorrelated with the transcripts level, indicating the involvement of post-transcriptional, translational and/or metabolic flux regulative processes. Can synthesis in TI-W after transferring it to 39 °C reached similar levels to those detected in C-W, although small differences in the distribution profile of the remaining carotenoids were present (Figure 3c). These were possibly associated with the influence of the growth temperature (39 °C) as inferred from the higher Ech levels in the temperature-inducible strain with respect to C-W (28 °C). Also, in TI-W a small but significant accumulation of Asx (~8%) occurred at 39 °C. For intermediate expression levels of CrtW (33 °C, Figure 3b), the Can relative content was approximately halved with respect to C-W and TI-W at 39 °C. This decrease in Can abundance was accompanied by an increase of Ech, consistent with a metabolic flux redistribution towards the “Ech/Can branch” that depended on the level of exogenous CrtW expression. Differently from the C-Z strain, in which the maximal Zea accumulation was more than 50% lower than in TI-Z at 39 °C, the C-W strain yielded equivalent levels of both Can and, to a lesser extent, Asx with respect to TI-W at 39 °C. Thus, for the production of Zea in *Synechocystis* the temperature-inducible TI-Z strain appears the most promising, but requires a two-step cultivation circle involving a temperature upshift. Instead, for the accumulation of Can, the constitutive C-W strain allows, in principle, for even higher product synthesis than in the temperature-inducible counterpart, but employs a simpler and standardised cultivation protocol.

Much more significant differences in the carotenoid profile and maximal level of accumulations were instead observed for the co-expression of *CrtW* and *CrtZ*, depending both on the positional gene cloning order as well as the promoter system. The maximal level of Asx production (>50%, Figure 3c) was attained in the temperature-inducible strains at 39 °C, with TI-ZW (1.1 ± 0.2 mg/g DCW) being slightly more efficient than TI-WZ (0.9 ± 0.3 mg/g DCW) (Figure 4). The carotenoid profile of TI-ZW was actually rather similar for transferring between 33 °C and 39 °C, whereas in TI-WZ a much larger Asx accumulation occurred at 39 °C, concomitantly with relative decreases in Ech and, to a lesser extent, in the Can levels. The constitutive expressing C-ZW strain displayed a carotenoid pattern that also was very similar to both TI-strains at 39 °C. On the other hand, the C-WZ strain showed much lower levels of Asx accumulation, as its carotenoid distribution appeared to be intermediate between C-W and TI-WZ at 33 °C, with Can being the principal carotenoid. All these differences highlight the importance of the positional cloning effect with respect to the promoter. In all the WZ strains, the limitation to the accumulation of Asx seemed to be somehow related with the expression/functionality of CrtZ. This is in part supported by the RT-PCR analysis of the C-ZW and C-WZ strains (Figure S3), which showed that the transcript of the first gene of the synthetic tandem is always more abundant than the following one. These results are briefly discussed in the Supplementary Materials section because of the already discussed general lack of correlation between the transcript levels and the carotenoid accumulation pattern. Another factor that might play an important role is the strength of the ribosome binding site (RBS) that has been interposed between the two genes, in the *CrtW*-RBS-*CrtZ* with respect to the *CrtZ*-RBS-*CrtW* cassette, as RBS activity can strongly depend on the sequence context [32,33]. 

Taken together, for biotechnological purposes targeting Asx biosynthesis, it can be concluded that the ZW ordering is by far the most promising and that the stable and temperature-inducible strains yield approximately similar levels of compound accumulation. The higher efficiency of the CrtZ-CrtW ordering is fully consistent with what was reported for exogenous Asx accumulation in other host organisms [31,34]. In *Synechocystis*, the highest Asx production yield was attained in TI-ZW at 39 °C (1.1 ± 0.2 mg/g DCW), therefore requiring a two-step procedure, which might represent a complication for large-scale cultivations. Thus, in principle, the constitutive expressing strains might be the most interesting for applicative purposes aimed at both Asx (C-ZW) and, as discussed above, Can (C-W) biosynthesis because these strains should allow simple continuous cultivation methodologies.

**Figure 5:**
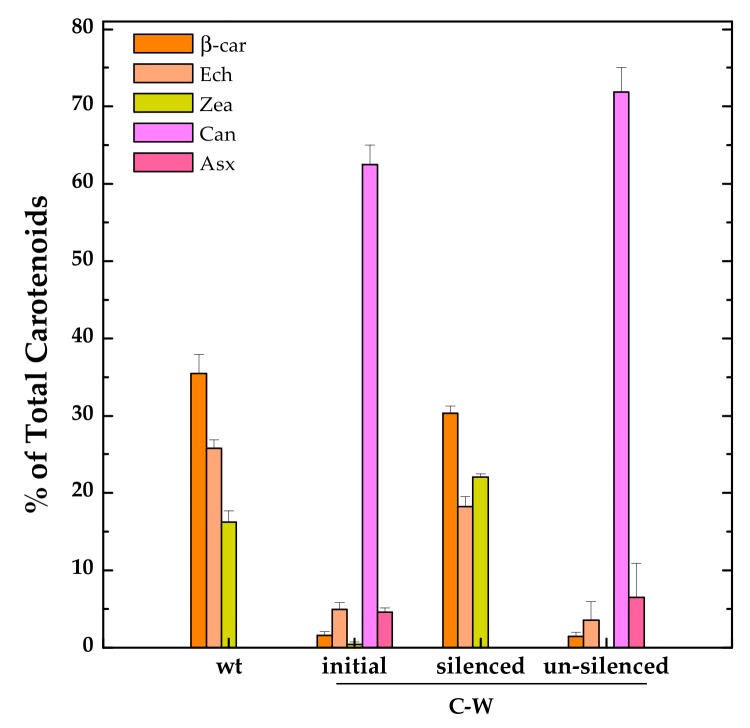
Relative distribution of the main carotenoids in the C-W strain constitutively expressing *Brevundimonas* CrtW shortly after its generation (“initial” phenotype), after several months of continuous sub-culturing in fresh medium (“silenced” phenotype) and grown to late stationary phase (“un-silenced” phenotype). Also shown is the carotenoid pattern of an exponentially growing wild-type (wt) culture for comparison. Ech, echinenone (peach); Zea, zeaxanthin (yellow); Can, canthaxanthin (light magenta); Asx, astaxanthin (pink). Error bars indicate SE.

However, we noticed, at least for one of these strains, C-W, some issues that might partially limit its biotechnological potential. Already during the initial phases of transformants selection, it was clear that the constitutive expression of CrtW was exerting a negative impact on cell growth. For strains carrying the *CrtW* gene (alone or in combination with *CrtZ*), only a few positive colonies could be isolated and their growth rate was reduced when compared with both the CrtZ expressing strain (C-Z) and the wild-type. The fact that full segregation could not be attained in these strains also hints at detrimental effects brought about by CrtW expression and/or the perturbation of the endogenous carotenoid biosynthetic pathway. In this respect it was observed that, after six months of continuous sub-culturing of the C-W strain, its ability to produce the non-endogenous keto-carotenoid Can was almost suppressed and the strain phenotypically reverted to a carotenoid profile resembling the wild-type one (Figure 5). 

Similar phenomena are commonly observed, but much less often reported in the literature nor investigated in detail [35]. Very little is known regarding genome instability in engineered cyanobacteria, but their extremely efficient DNA recombination and repair machinery [36] is certainly involved, enabling them to inactivate the newly introduced genes if this will threaten cell fitness by creating insertions, deletions or replacement mutations. These DNA modifications are generally irreversible. In the case of C-W, the phenotypically observable “silencing” was instead transitory since cultures kept growing in liquid to over-reach the stationary phase recovered their capability to accumulate Can at impressive levels, up to ~70% (Figure 5), even exceeding that of the same strain analysed shortly after its generation, and sampled during the exponential growth phase. The exact mechanism by which Can accumulation was transiently inactivated in C-W is still unclear, but seems to be somehow related to the physiological state of the culture. Since this represents a fascinating observation, which is also relevant for the biotechnological potential of constitutive producing strains, it certainly deserves further investigation in the near future. 

## 5. Conclusions

Two different approaches for the heterologous expression of the carotenogenic enzymes CrtW and CrtZ from *Brevundimonas* were employed for keto-carotenoid production in *Synechocystis*. For the constitutive expression, a strong native promoter was chosen, whereas conditional expression was obtained through a temperature-inducible promoter system. Although significant accumulation of non-endogenous keto-carotenoids was reached in both cases, the maximum level of the specific carotenoid of interest depended on the genetic manipulation strategy applied. The highest level of Can accumulation (1.3 ± 0.1 mg/g DCW) was attained in the strain constitutively expressing CrtW (C-W). However, C-W showed some production instability depending on the age of the culture that did not affect the temperature-inducible TI-W strain instead. On the other hand, the most efficient strain for Asx production was the temperature-inducible strain TI-ZW (1.1 ± 0.2 mg/g DCW at 39 °C), which yielded satisfactory amounts of this compound already at 33 °C (1.0 ± 0.2 mg/g DCW). Although temperature-inducible strains have the drawback of requiring two sequential cultivation periods, they allow for microbial growth to high cell density before switching on the exogenous biosynthetic pathway. In this way the endogenous “defence mechanisms” aimed at maintaining cell viability during the grow phase can be bypassed, or their effects minimised, making it possible to attain high accumulation levels of substances that might exhibit cytotoxic activity or lower the cell fitness.

## Supplementary Materials

The following are available online at https://www.mdpi.com/2076-2607/7/11/501/s1.
