# Supplementary material for: A Comparison of Constitutive and Inducible Non-Endogenous Keto-Carotenoids Biosynthesis in Synechocystis sp. PCC 6803
Source: Microorganisms. 2019 Oct 28;7(11):501. doi: 10.3390/microorganisms7110501 (PMC6920976; doi:10.3390/microorganisms7110501)
Supplement: Supplementary file 1 [file microorganisms-07-00501-s001.pdf]

Supplementary Materials:

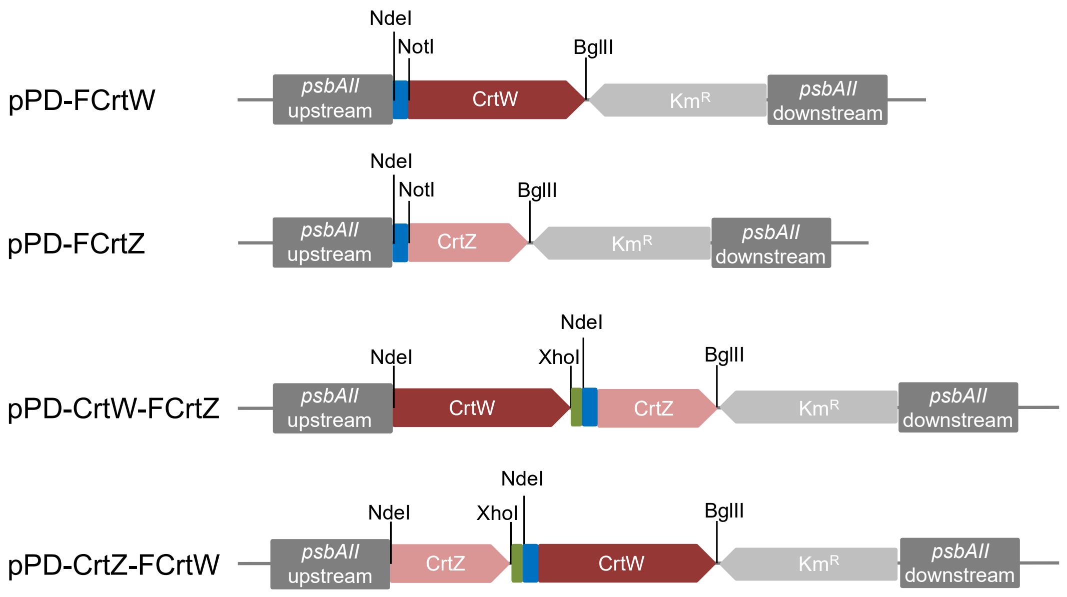

Figure S1: Schematic representation of generated plasmids. Sequences corresponding to *Synechocystis* genomic regions located immediately up- and down-stream of the *psbAII* gene, which promote homologous recombination between the plasmid and the *Synechocystis psbAII* gene, are indicated by grey boxes. *psbAII* upstream region comprises the strong native *psbAII* promoter. Also indicated are the Km^R gene (light grey arrow), the 3xFLAG tag (blue box) and the ribosome binding site (green box).

Table S1. List of primers used in this study.

Name	Sequence 5'—3'	Purpose
CrtW_5'NotI	AAGGAAAAAAGCGGCCGCAGCCGT GGCTGAACCCCGTATT	Forward primer to amplify synthetic CrtW with a NotI site[1]
CrtW_3'BglII	GAAGATCTTAACTTTCGCCACGCCAC	Reverse primer to amplify synthetic CrtW with a BglII site[1]
CrtZ_5'NotI	AAGGAAAAAAGCGGCCGCAGCCTG GTTGACCTGGATTGCC	Forward primer to amplify synthetic CrtZ with a NotI site[1]
CrtZ_3'BglII	GAAGATCTTAGGCCCCACTGGAAC	Reverse primer to amplify synthetic CrtZ with a BglII site[1]
PsbAII_up	TGAACATCGACAAATACATAAG	Forward primer inside the psbAII upstream region (starting 35 bp upstream of the *psbAII* ATG) to check for chromosomal insertion and segregation
PsbAII_do	ATTATTCAGTTGGCATTACACC	Reverse primer inside the psbAII downstream region (starting 31 bp downstream of the *psbAII* stop codon) to check for chromosomal insertion and segregation
CrtW_F	GCACCGCCGATGATCCC	Forward primer inside synthetic CrtW to check for its correct orientation in pPD-CrtW-FCrtZ
CrtZ_F	CGTGGGCTTAGGTATTACC	Forward primer inside synthetic CrtZ to

		check for its correct orientation in pPD-CrtZ-FCrtW
CrtW_R	GGGATCATCGGCGGTGC	Reverse primer inside synthetic CrtW to check for CrtZ correct orientation in pPD-CrtZ-FCrtW, for chromosomal insertion and segregation
CrtZ_R	GGTAATACCTAAGCCCACG	Reverse primer inside synthetic CrtZ to check for CrtW correct orientation in pPD-CrtW-FCrtZ, for chromosomal insertion and segregation

[1] Inserted restriction sites are underlined.

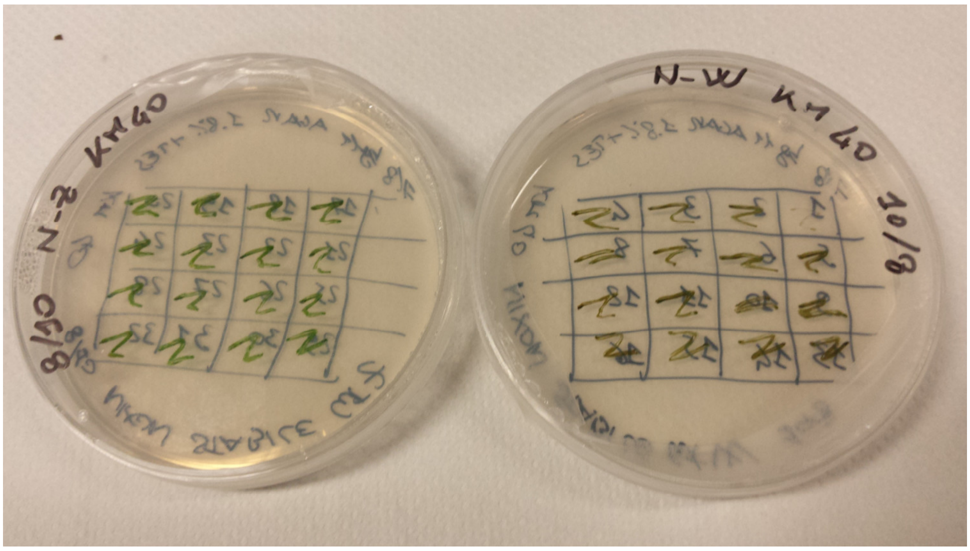

Figure S2: Colour appearance of transformants during selection. Comparison between several colonies (16) constitutively expressing CrtZ (N-Z) and CrtW (N-W) from *Brevundimonas* that were peaked and re-plated on selective medium containing 40 µg/ml Kanamycin, evidencing the different pigmentation.

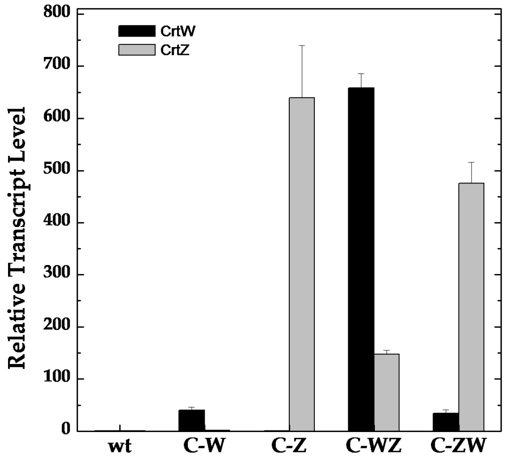

Figure S3: Expression analysis of *CrtW* (black bars) and *CrtZ* (grey bars) in the engineered C-strains. Transcript abundance was quantified using qRT-PCR and expression levels were normalised to *rnpB* (wild-

type was used as calibrator). Data are the means of three qRT-PCR technical replicates for each biological duplicate, error bars indicate SE.

qRT-PCR analysis of the C-strains indicates a general lack of correlation between the transcript levels of *CrtW* and *CrtZ* and the carotenoid accumulation pattern. *CrtZ* is highly expressed in C-Z, but the observed increase in Zea content is relatively low (a ~30% increment with respect to the wild-type). *CrtW* is expressed at rather lower levels in C-W, despite being under the control of the same strong promoter as *CrtZ* in C-Z, but the accumulation of Can was the highest (more than 60% of the total pool). In C-WZ the relative abundance of the *CrtZ* transcript is several folds lower than the one of *CrtW*. If there were to be a simple correlation between gene transcription and product accumulation, this would explain the very similar carotenoid pattern observed in C-WZ and C-W. However the *CrtW* expression level in C-W was rather low. Moreover, in C-ZW, despite *CrtW* transcription being several fold lower than *CrtZ*, the resulting carotenoid pattern phenotype was completely different from that of C-Z, and indeed C-ZW is amongst the strains accumulating the larger Asx amounts. It is arguable that the low expression of *CrtW* in this strain is sufficient for the production of exogenous keto-carotenoids, but at the same time highlights the need for further detailed investigations at the protein level, to achieve a better understanding of the observed phenotypes.

Experimental procedure: total RNA was extracted from each strain in the presence of NucleoZOL (Macherey-Nagel) following the manufacturer instructions. RNA integrity was checked by agarose gel electrophoresis. The absence of contaminant DNA was checked by PCR using primer specific to the control gene *rnpB* (RT-rnpB_F 5′- CGTTAGGATAGTGCCACAG-3′ and RT-rnpB_R 5′- CGCTCTTACCGCACCTTTG-3′), which was also used as endogenous control in qRT-PCR. cDNA synthesis was performed from 1.3 µg of DNA-free RNA. Reactions were run in the 7300 RealTime PCR System (Applied Biosystems) and data analysed using the 7300 System Software (Applied Biosystems). The following primers were used: for CrtW, RT-CrtW_F 5′- ATTGCCTTATTTGGGTTGGG-3′ and RT-CrtW_R 5′- CAGGTGCCAAAGGTAAACAA-3′; for CrtZ, RT-CrtZ_F 5′- GGATTATGCACCGTTATGTG-3′ and RT-CrtZ_R 5′- CACGGCAAATAAATCATTCT-3′. For each gene, cDNA dilution curves were generated (cDNA dilutions: 1/3, 1/9, 1/27 and 1/81) and used to calculate the individual real-time PCR efficiencies. Reactions were manually assembled and contained 0.25 µM of each primer, 10 µL of iTaq Universal SYBR Green Super mix (Bio-Rad) and 2 µL of template cDNA (dilution 1/27). The PCR program consisted of: 3 min at 95°C followed by 40 cycles of 30 s at 95°C, 30 s at 56°C and 30 s at 72°C. Dissociation analysis was performed at the end of each run to confirm the specificity of the reaction. Quantitative variation was evaluated by the $2^{-\Delta\Delta CT}$ method.
